# A Snapshot of Poverty, Diseases and war – the Democratic Republic of the Congo

**DOI:** 10.1017/dmp.2021.227

**Published:** 2021-07-15

**Authors:** Shibu Sasidharan, Harpreet Singh Dhillon

**Affiliations:** 1 Department of Anaesthesiology and Critical care, Level III IFH, MONUSCO, Goma, DRC; 2 Psychiatry, Level III IFH, MONUSCO, Goma, DRC

**Keywords:** COVID-19, Measles, Ebola, Rift Valley fever

## Abstract

DRC’s fight with the EVD (Ebola Virus Disease) was just settling when WHO declared COVID-19 to be a Public Health Emergency of International Concern (PHEIC) on March 12, 2020. DRC’s economic growth decelerated from its pre-COVID level of 4.4% in 2019 to an estimated 0.8% in 2020. This has caused concomitant setbacks in the treatment and control of major health issues like HIV, tuberculosis, measles, rift valley fever and malaria in the country. This, coupled with civil unrest, other infectious diseases and risk to the safety of the health workers is a recipe for a ‘perfect storm’ waiting to unfold.

## A Snapshot of Poverty, Diseases and War – The Democratic Republic of the Congo

The Democratic Republic of the Congo (DRC) is a country located in Central Africa, and has a population of about 69.6 million people. DRC is endowed with exceptional natural resources, including minerals such as cobalt and copper, hydropower potential, significant arable land, immense biodiversity, and the world’s second largest rainforest. Poverty in DRC is high, remains widespread and pervasive, and is increasing due to impact from COVID-19. In 2018, it was estimated that 73% of the Congolese population, equaling 60 million people lived on less than $1.90 a day (the international poverty rate). As such, about 1 out of 6 people living in extreme poverty in Sub-Saharan Africa (SSA) live in DRC. With a GDP per capita of only $753, the DRC is 1 of the poorest countries in the world with half the country’s population below the poverty line. Life expectancy at birth for females is 61 and for males, 58 years. With an infant mortality rate as high as 67/1000 live births,^1^ the problems faced by DRC are more unique than other parts of Africa. DRC’s Human Capital Index is 0.37%, below the Sub-Saharan Africa (SSA) average of 4.0. This means that a child born in DRC today will be 37% as productive in adulthood as she could be if she enjoyed complete education and full health in her early years. On average, a Congolese child receives 9.1 years of schooling, which translates into 4.5 years of learning-adjusted years of school (estimate for the year 2020). In addition to all of this, 43% of children are malnourished,^2^ here lies a story of tremendous abandonment that needs to be told.

Neglected tropical diseases cause substantial illness for more than 1 billion people globally. Many diseases like diarrhea and cholera in the DRC stem from basic inadequacies like a lack of adequate sanitation and safe water. In the DRC, less than 25% of people have access to clean water, this results in a high probability of contracting water borne diseases like Cholera which has frequent outbreaks.^3^ Rare diseases like Onchocerciasis, also known as river blindness, a disease caused by a parasitic worm and spread by an infected fly is also 1 of the many major health issues.^4^

The Democratic Republic of the Congo (DRC) has the second highest number of malaria cases and deaths globally, 12% of cases and 11% of deaths respectively, and 54.6% of the malaria cases in Central Africa as at 2018 which accounts as a major cause of morbidity and mortality. In 2019 alone, more than 13000 deaths were reported from malaria in the DR Congo with children younger than 5 years of age accounting for 67% of deaths.^5^ The World Health Organization (WHO) has set 2030 as the deadline for malaria elimination. The various measures adopted for the same include providing long-lasting insecticide-treated nets (LLIN), preventing malaria in pregnancy, improving diagnostics and case management, surveillance and monitoring, and the evaluation of malaria-related activities.

HIV is a leading cause of death and also a health threat to millions worldwide. Key populations for HIV include: men who have sex with men, people who inject drugs, people in prisons and other closed settings, sex workers and their clients, and transgender people. They are at an increased risk of being infected with HIV irrespective of epidemic type or local context. In Africa, key populations, their clients and sexual partners accounted for 64% of the new HIV infections in West and Central Africa, and for 25% of the new HIV infections in the Eastern and Southern sub region of Africa.^6^ With the goal to build a sustainable, high-impact national HIV response program to accelerate progress towards the UNAIDS global target to control the epidemic, many international organizations have made significant contributions for the same cause.

Measles is a major concern in the DRC. According to the WHO, DRC has witnessed the world’s worst measles epidemic with 369520 cases and 6779 deaths since 2019 attributable to poor vaccination coverage especially in remote areas, malnutrition, inaccessible geographical terrain, frail public health infrastructure, weak political will, and outbreaks of other epidemics.^7^

**Figure 1.**
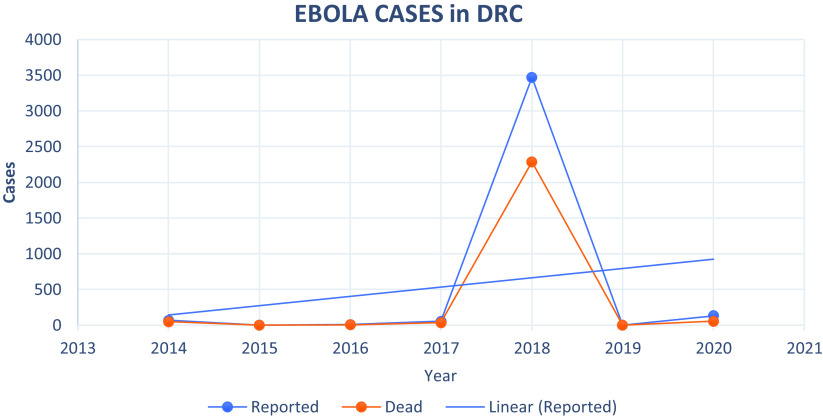
Ebola cases in DRC.

The Ebola Virus Disease (EVD) raged through Africa between the years 2014 to 2016, and it went on to be the biggest International Public Health Concern of International Concern (PHEIC) of the decade (Figure 1). On May 14, 2020, the Ministry of Health began the 42-day countdown to the declaration of the end of that outbreak. Cases had dropped to 0 by Feb 17, 2020, but after 52 days without a case, surveillance and response teams on the ground confirmed 3 new cases of Ebola in Beni health zone in mid-April raising concerns of an animal reservoir. In 2020 there were 130 reported cases of EVD with a fatality of 55 cases.^8^

**Figure 2.**
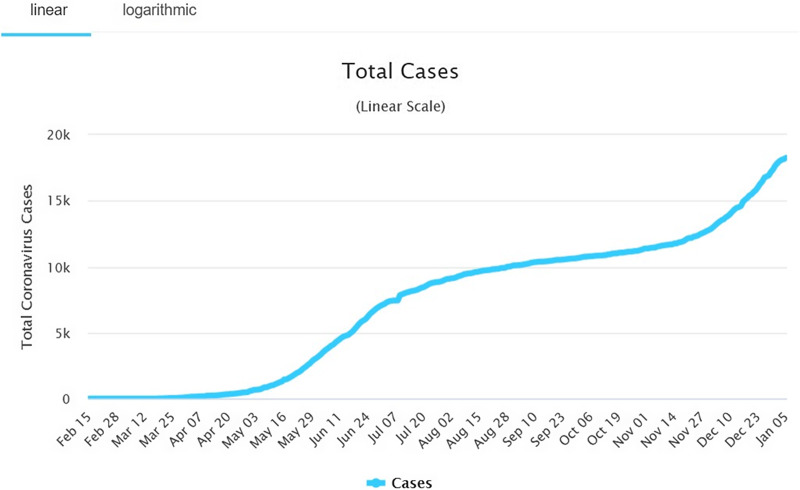
Total cases of Coronavirus in DRC.

DRC’s fight with EVD was just settling when the WHO declared COVID-19 to be a PHEIC on March 12, 2020. Government authorities in DRC have placed various measures like systematic body temperature screening, banning all public gatherings, and suspending all flights and air traffic since March 19, 2020. Borders were closed and a state of emergency was declared on March 24. Despite these measures, the virus continued progressing throughout the country, which suggests that these measures were inadequate. As at May 2020, 18247 cases were reported with 599 deaths (Figure 2).^9^

Rift Valley fever (RVF) is another life-threatening disease that affects the residents of DRC. It is a zoonotic mosquito-borne disease caused by the RVF virus (RVFV) which causes abortions and a high mortality rate in livestock, and is also associated with acute and fatal disease in humans. In the Democratic Republic of the Congo (DRC), information on the epidemiology of RVF, particularly from cattle reared by smallholder farmers indicate that cattle in a few provinces had been exposed to RVFV, which represents a significant risk for both livestock and human health.^10^ People can get RVF through contact with blood, body fluids, or tissues of infected animals. This direct contact can occur during slaughter or butchering, while caring for sick animals, during veterinary procedures like assisting an animal with giving birth, and when consuming raw or undercooked animal products. People can also get RVF through bites from infected mosquitoes and rarely, from other insects that bite. Infection with the RVF virus (RVFV) has occurred in laboratories when someone inhaled the virus that was in the air (known as aerosol transmission). The spread from person to person has not been documented yet, and no transmission of RVF to health care workers has been reported when standard infection control precautions have been put in place. The incubation period (the interval from infection to onset of symptoms) for RVF varies from 2 to 6 days.^11^ Although RVF often causes severe illnesses in animals, most people with RVF either have no symptoms, or a mild illness with fever, weakness, back pain, and dizziness. However, a small percentage (8 - 10%) of people with RVF develop much more severe symptoms, including eye diseases, hemorrhage (excessive bleeding), and encephalitis (swelling of the brain).^12^ Outbreaks of RVF in animals can be prevented by a sustained program of animal vaccination.^11^

Between 1997 and 2003, the country was engulfed in a civil war. The war’s central cause was a desire for possession over the DRC’s mineral wealth, water and food which gravely dented its infrastructure. Violence due to political instability makes it difficult for aid workers to access the area. Additionally, a multitude of diseases devastate the nation, common of which are malaria, HIV, diarrheal diseases from lack of clean water, measles and cholera.^13^

## Recommendations

The following recommendations can be executed in order to curb the uncontrolled wave of disease:

1. Strengthening and scaling up of existing public health systems: The existing public health system needs to be strengthened and scaled up by enhancing laboratory capabilities and recruitment of the health care workforce.
2. Comprehensive and integrated response to HIV and Tuberculosis (TB): The prevention, testing, and treatment strategies for both HIV and TB should be integrated. The 5 key populations defined by the WHO and UN partners deserve particular and specific attention. In the African region, these populations with HIV face structural barriers to services that compound their risk exposure – barriers such as laws that criminalize their behavior, stigma, discrimination and violence.
3. IEC (information, education and communication) activities: The general public needs to be sensitized regarding infection prevention, vaccination, and the dissemination of public health information to remote areas.
4. Epidemiological investigations: For this, a meticulous data collection, and compilation of line-lists and analysis of outbreak investigations are recommended to define the epidemiology of the epidemic, and to guide quick and operative reactive campaigns. Population-based coverage surveys should be implemented to determine the susceptibility profile, and to recognize spaces of low coverage to better prioritize and resourcefully use capitals in order to target the most vulnerable groups. Based on the data collected, a triage tool can be designed akin to Pandemic influenza Triage tool (PITA) developed by CDC for Influenza.^14^ Such a triage tool can further be integrated with existing community healthcare decision making tools to expedite management of patient surge at community level during Pandemics.^15^
5. National response for containment: The ministry of health and local government resources should have standard operating procedures in place for strict border lockdowns, to quickly and effectively help prevent the regional spread of diseases as well as around the world.
6. Field Epidemiology Training Program (FETP): The establishment of the FETP for strengthening the capacity of its workforce to investigate and respond to disease outbreaks is an effective tool developed by CDC. FETP trains field epidemiologists, or disease detectives to identify and contain outbreaks before they become epidemics. Participants focus on ‘learning by doing’ to develop the skills for gathering critical data and turning it into evidence-based action. The DRC FETP has trained 196 disease detectives who are crucial to accurately detecting and identifying outbreaks, including the recent Ebola outbreaks. The first cohort graduated in 2014 and helped support the responses to the 2014, 2017, and 2018 Ebola outbreaks. A total of 148 graduates have rotated to support the Ebola response in the North Kivu province.
7. Global Health Security: Helping countries respond to public health threats quickly and effectively within their borders is critical to preventing the spread of diseases regionally, and around the world. International health bodies like the CDC can collaborate with the MOH and other partners in the DRC to support disease outbreak response, surveillance, laboratory systems, and workforce development.
8. Outbreaks of RVF in animals can be prevented by a sustained program of animal vaccination, public health education and risk reduction, vector control, and infection control in health care settings.
